# Kinematic alignment in total knee replacement successfully restores the native knee phenotype in the short term

**DOI:** 10.1002/jeo2.70561

**Published:** 2025-11-14

**Authors:** Mohammad Mahdi Sarzaeem, Mohammad Movahedinia, Ali Saeidi, Mohammadsajjad Sarzaeem, Mohammad Mahdi Omidian, Salar Baghbani, Rasoul Shirmohammadi, Yashar Shahbaz

**Affiliations:** ^1^ Department of Orthopedic Surgery and Traumatology Shahid Beheshti University of Medical Sciences Tehran Iran; ^2^ Second Faculty of Medicine Charles University Prague Czechia; ^3^ Orthopedic Surgery Ward, Besat Hospital, Trauma and Surgery Research Center Aja University of Medical Sciences Tehran Iran; ^4^ Department of Orthopedic Surgery, Sina Hospital, School of Medicine Tehran University of Medical Sciences Tehran Iran; ^5^ Orthopedic Research Center, Shahid Kamyab Hospital, School of Medicine Mashhad University of Medical Sciences Mashhad Iran

**Keywords:** ethnic variation, gender differences, kinematic alignment, knee phenotypes, population‐specific alignment, total knee arthroplasty

## Abstract

**Purpose:**

The goal of kinematic alignment (KA) in total knee arthroplasty (TKA) is to bring back the natural knee phenotypes. This study compared postoperative functional phenotypes in KA‐TKAs against native phenotypes of healthy young Iranians, addressing the need for population‐specific alignment targets.

**Methods:**

The study analysed 300 KA‐TKAs and 150 native phenotypes of healthy young Iranians. Full‐limb radiographs were used to measure alignment parameters and classify phenotypes. Patient‐reported outcomes were collected to assess functional recovery. The research focused on phenotype restoration, gender differences and ethnic variations in constitutional alignment.

**Results:**

KA‐TKA successfully restored constitutional knee phenotypes in 91.7% of patients. The top five phenotypes showed similar proportions in both TKA (52.7%) and control (54.2%) groups. Females demonstrated higher phenotype restoration rates, particularly for valgus‐dominant phenotypes. The Forgotten Joint Scores at 3 months showed a significant improvement of 18% when comparing phenotype‐matched patients to outliers. KA‐TKA cases that failed to match the control phenotype distribution amounted to 8.3%, and these cases showed significant preoperative deformities as well as high body mass index. The research demonstrated important differences in ethnic populations since 53.7% of Iranians showed constitutional varus compared to 28.3% of Swiss individuals.

**Conclusion:**

The study demonstrates that KA‐TKA successfully restores native knee phenotypes to most Iranian patients. The alignment targets require customization for population‐specific standards together with attention to gender‐specific variations.

**Level of Evidence:**

Level III.

**Abbreviations**
FMA: femoral mechanical angle
HKA: hip–knee–ankle angle
ICC: intraclass correlation coefficient
JLCA: joint line convergence angle
KA: kinematic alignment
MA: mechanical alignment
mLDFA: mechanical lateral distal femoral angle
mMPTA: mechanical medial proximal tibial angle
OA: osteoarthritis
TKA: total knee arthroplasty
TMA: tibial mechanical angle

## INTRODUCTION

The projected number of total knee arthroplasty (TKA) surgeries during 2025 will exceed 2005 numbers by 673% which shows its essential function for treating knee osteoarthritis (OA) in the worldwide elderly population [6]. Since its inception mechanical alignment (MA) has served as the primary philosophical approach for TKA by establishing neutral hip–knee–ankle angle (HKA) at 180° through perpendicular component positioning to mechanical axes [5]. This method concentrates on creating long‐lasting implants that reduce uneven loading forces [29]. However, recent evidence suggests a significant limitation when MA‐TKA ignores the anatomical variability of the natural knee [3]. Studies demonstrate that only 3.6%–5.6% of young, nonosteoarthritic knees exhibit the ‘neutral’ phenotype targeted by MA‐TKA, while anatomical phenotypes vary widely across ethnicities and sexes [12, 13, 28]. Consequently, MA‐TKA often creates nonphysiological joint lines, leading to soft‐tissue imbalances, aberrant knee kinematics and patient dissatisfaction rates of 7%–20% despite technically successful surgeries [6, 7, 8]. This gap between the attempt to zero out HKA and the anatomical reality of the body has accelerated the shift in trends towards the kinematic alignment (KA) method [27]. Research on bilateral symmetry demonstrates that only 26% of people show matching contralateral coronal phenotypes [22], which indicates that KA should focus on single‐side anatomy instead of copying the unaffected knee.

The introduction of KA as a revolutionary alternative to MA promoted the restoration of prearthritic coronal, sagittal and axial joint line orientations instead of imposing universal neutral axis conformity [16, 24]. KA‐TKA protects native soft‐tissue tension and articular surface geometry through minimal bone resections and ligament releases because it honours individual anatomy [6, 27]. Theoretically, this enhances functional outcomes, proprioception and patient satisfaction by replicating physiological knee biomechanics [27]. However, the effectiveness of KA‐TKA depends on accurately restoring the patient's prearthritic functional phenotype, a concept that Hirschman et al. incorporated into their classification system [12, 19]. This classification characterizes coronal alignment through three interrelated angles: the HKA, the femoral mechanical angle (FMA) and the tibial mechanical angle (TMA). Phenotypes are defined by 3° variations from each other and clearly reflect the variation observed in nonarthritic populations [12, 13]. Global studies show major ethnic differences together with sex‐based distinctions in these phenotypic patterns. Research indicates that the majority of Iranian subjects show neutral to mild varus knee alignment (coronal plane alignment knee [CPAK] Type I: 33.6%, Type II: 26.6%) while female participants have more tibial valgus (41.6% valgus TMA) than male participants (62.8% neutral TMA) [23]. The observed variations demonstrate that applying a uniform alignment approach to everyone remains theoretically unacceptable.

Although KA‐TKA shows theoretical advantages, important questions about its effectiveness remain unanswered: Does KA truly regain the functional characteristics that are typical in healthy nonarthritic individuals? Most KA‐TKA outcome studies rely on traditional alignment categories (neutral/varus/valgus), neglecting the practical classification established by Hirschmann et al. and the diversity of FMA–TMA–HKA interactions, which define physiological joint function [10, 13, 23]. Studies confirm that Iranian knees, as a populous part of the Middle East, differ from European groups and show a higher prevalence of varus and sexual dimorphism [23], but no study has evaluated whether KA restores healthy alignment in this population.

Our primary hypothesis was that KA‐TKA can restore postoperative phenotypes to the healthy Iranian adults' limb alignments. Secondary aims included correlating phenotype‐matched alignment with patient‐reported outcomes, providing evidence for phenotype‐driven TKA personalization.

## MATERIAL AND METHODS

Patient recruitment took place between January 2019 and December 2024 at a tertiary orthopaedic centre in Iran. The research implemented a matched‐pair design which compared two different groups:

1. TKA cohort containing 300 consecutive patients with end‐stage OA who underwent KA‐TKA. The study selected primary OA patients with Kellgren–Lawrence Grade 3 or higher and who had exhausted conservative treatment (nonsteroidal anti‐inflammatory drugs [NSAIDs] and physical therapy for at least 6 months) and exhibited coronal deformity of 20° or less in standing radiographs. Inflammatory arthritis (e.g., rheumatoid arthritis), prior knee osteotomy or ligament reconstruction and neuromuscular disorders affecting gait were considered as exclusion criteria. All TKAs were performed by a single surgeon using a standardized unrestricted KA protocol [25].
2. Control cohort including 150 asymptomatic healthy Iranian adults (aged 18–35 years) representing the nonarthritic knee phenotype distribution without any history of knee injury or radiographic OA (Kellgren–Lawrence Grade 0). More than three degrees of discrepancy between the lower limbs alignment was considered as an exclusion criterion.

Sample size calculation was based on Hirschmann's phenotype prevalence data [12], ensuring 90% power (*α* = 0.05) to detect a 15% intercohort phenotype distribution difference.

To ensure the accuracy and reproducibility of all radiographic measurements, a standardized imaging protocol was used for all participants both preoperatively and 3 months post‐TKA. Participants were positioned in a standardized stance according to the established protocol for alignment assessment [18]. Specifically, the feet were placed shoulder‐width apart in a neutral rotation position, confirmed by ensuring the patellae were facing directly forward (‘patella‐forward' position). Patients were instructed to distribute their weight equally on both limbs, and full knee extension was verified by the technologist. This rigorous protocol minimizes the confounding effects of limb rotation and stance on the measurement of the HKA, mechanical lateral distal femoral angle (mLDFA), mechanical medial proximal tibial angle (mMPTA) and joint line convergence angle (JLCA), thereby ensuring the reliability of the subsequent phenotypic classification. Radiographic assessments were conducted by two independent orthopaedic surgeons who were blinded to the cohort assignments. Phenotype classification was performed using Hirschmann's system (Table 1).

**Table 1:** Hirschmann phenotype classification framework.

Parameter	Definition	Categories
HKA phenotype	Coronal limb alignment	NEU, VAR, VAL (3° increments)
Femoral phenotype	mLDFA relative to mechanical axis	NEU, VAR, VAL (3° increments)
Tibial phenotype	mMPTA relative to mechanical axis	NEU, VAR, VAL (3° increments)
Functional phenotype	Combined HKA, femoral and tibial classifications	43 unique phenotypes (e.g., NEUHKA0°+NEUFMA0°+NEUTMA0°)

Abbreviations: HKA, hip–knee–ankle angle; mLDFA, mechanical lateral distal femoral angle; mMPTA, mechanical medial proximal tibial angle.

Furthermore, Knee Injury and Osteoarthritis Outcome Score (KOOS) and Forgotten Joint Score‐12 (FJS‐12) were gathered from all participants including preoperatively and 3 months post‐TKA.

SPSS v28.0 (IBM Corp.) was used with significance set at *p* value < 0.05. Phenotype distribution was compared via *χ* ^2^ tests and Cramer's *V* for effect size. Paired *t*‐test was used for pre‐ and post‐TKA alignment comparison. Multivariable regression was used to identify predictors of phenotype restoration (age, body mass index [BMI], preoperative HKA). Intraclass correlation coefficient (ICC) was used for radiographic measures (two blinded observers).

## RESULTS

The study included 300 patients who underwent KA‐TKA (mean age 68.2 ± 7.1 years, 64% females) and 150 healthy controls (mean age 28.4 ± 4.3 years, 58% females). Significant intercohort differences existed in age, BMI and preoperative coronal alignment (*p* < 0.001), while sex distribution was comparable (Table 2).

**Table 2:** Demographic and baseline radiographic characteristics.

Parameter	TKA cohort (*n* = 300)	Control cohort (*n* = 150)	*p* value
Age (years)	68.2 ± 7.1	28.4 ± 4.3	<0.001
Female	192 (64.0%)	87 (58.0%)	0.21
BMI (kg/m^2^)	29.5 ± 4.2	24.1 ± 3.0	<0.001
Preoperative HKA (°)	−6.8 ± 4.1 (varus)	−1.2 ± 2.0	<0.001
Preoperative JLCA (°)	2.8 ± 1.6	0.8 ± 0.9	<0.001

Abbreviations: BMI, body mass index; HKA, hip–knee–ankle angle; JLCA, joint line convergence angle; TKA, total knee arthroplasty.

Postoperative TKA phenotypes were not significantly different from healthy controls (*κ* = 0.87, *p* < 0.001). As shown in Table 3, the top five phenotypes accounted for 52.7% of TKA patients and 54.2% of controls, with no significant intercohort differences in prevalence (*p* = 0.31).

**Table 3:** Comparison of the most prevalent functional phenotype distribution between cohorts of the study.

Phenotype (HKA° | FMA° | TMA°)	TKA cohort (*n* = 300)	Control cohort (*n* = 150)	*p* value
177° | 90° | 87°	37 (12.3%)	19 (12.7%)	0.91
174° | 90° | 84°	33 (11.0%)	17 (11.3%)	0.94
174° | 87° | 87°	23 (7.7%)	11 (7.3%)	0.88
177° | 93° | 84°	23 (7.7%)	11 (7.3%)	0.88
171° | 87° | 84°	17 (5.7%)	8 (5.3%)	0.85
All other phenotypes	167 (55.7%)	84 (56.0%)	0.95

Abbreviations: FMA, femoral mechanical angle; HKA, hip–knee–ankle angle; TMA, tibial mechanical angle; TKA, total knee arthroplasty.

Females showed higher degree of restoration than males (*κ* = 0.92 vs. *κ* = 0.81), particularly in phenotypes with combined tibial valgus and femoral varus (e.g., 177° | 90° | 87°: 93.3% match). As shown in Table 4, Iranian females exhibited higher overall native varus or valgus alignment (*p* < 0.001).

**Table 4:** Gender‐specific phenotype distribution.

Phenotype (HKA° | FMA° | TMA°)	Female prevalence		Male prevalence	
	TKA	Control	TKA	Control
177° | 90° | 87°	12.0%	12.5%	6.5%	6.7%
174° | 90° | 84°	10.4%	10.7%	8.9%	8.0%
Valgus‐dominant	22.1%	21.8%	15.3%	16.1%

Abbreviations: FMA, femoral mechanical angle; HKA, hip–knee–ankle angle; TMA, tibial mechanical angle; TKA, total knee arthroplasty.

**Figure 1:**
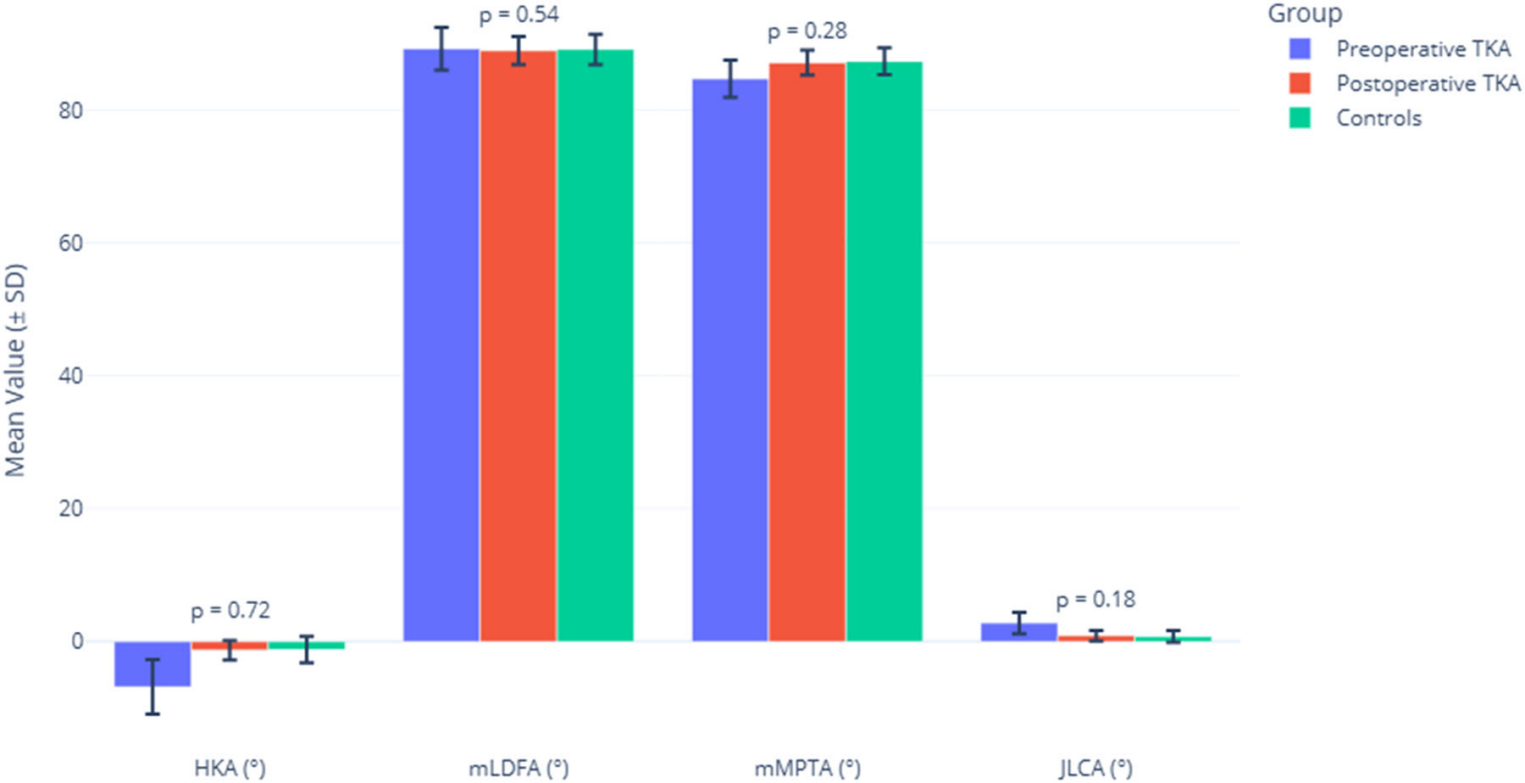
Radiographic alignment parameters: preoperative versus postoperative TKA versus controls. *p* values are between postoperative TKA and controls. TKA, total knee arthroplasty; HKA, hip–knee–ankle angle; mLDFA, mechanical lateral distal femoral angle; mMPTA, mechanical medial proximal tibial angle; JLCA, joint line convergence angle; SD, standard deviation.

Figure 1 shows that KA‐TKA effectively restored all parameters to control levels (*p* > 0.05 for all postoperative vs. control comparisons). JLCA normalized from 2.8° ± 1.6° to 0.9° ± 0.8° (controls: 0.8° ± 0.9°). Limb alignment improved from −6.8° ± 4.1° to −1.3° ± 1.5° (controls: −1.2° ± 2.0°).

**Figure 2:**
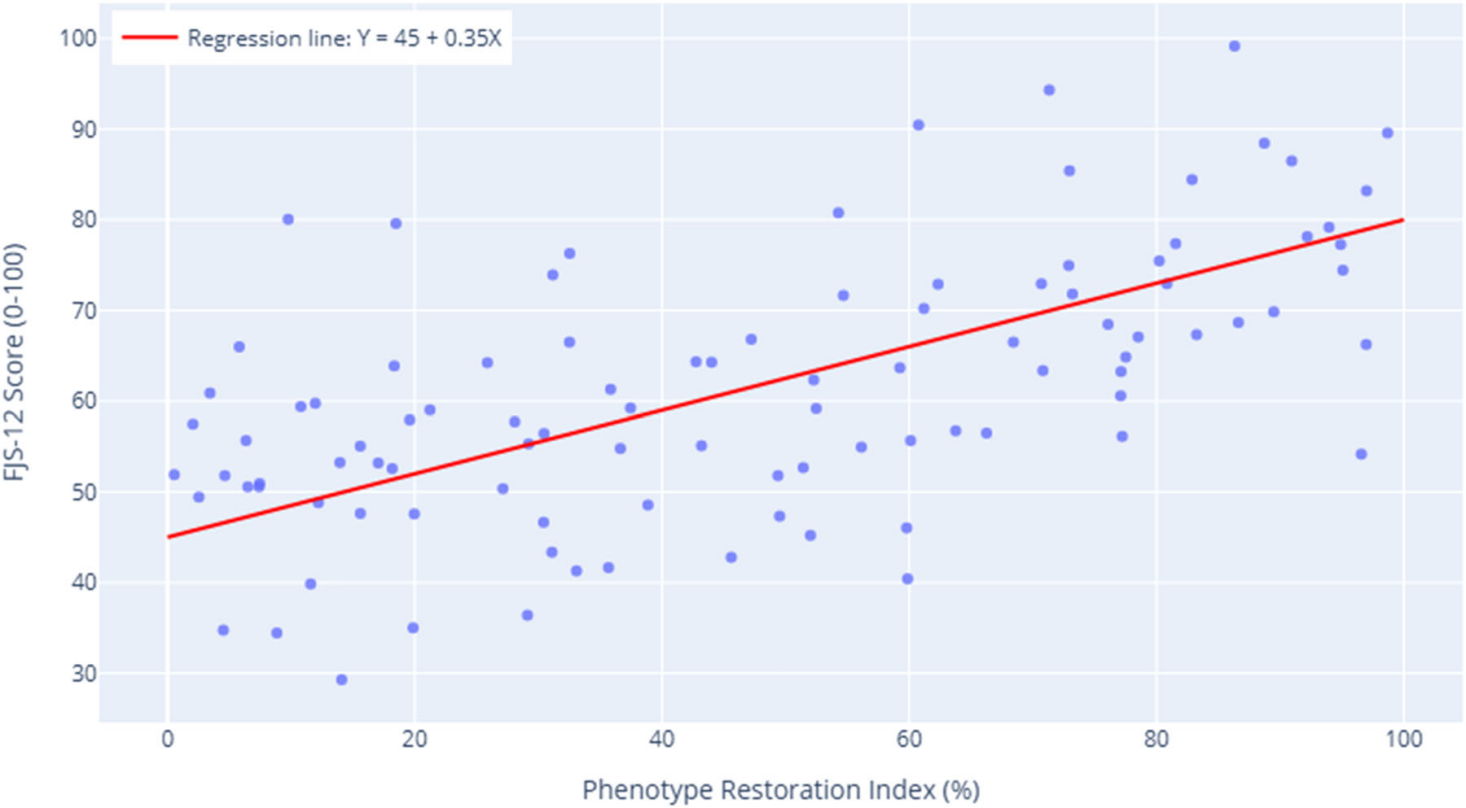
Functional outcome correlation with phenotype restoration. FJS‐12, Forgotten Joint Score‐12.

At the 3‐month follow‐up, KA‐TKA patients demonstrated excellent functional outcomes. KOOS‐Pain: 84.3 ± 9.2 (vs. 92.5 ± 5.1 in controls), FJS‐12: 68.7 ±12.4 (72% achieving ‘forgotten joint’ threshold >60). Phenotype‐matched patients showed 18% better FJS‐12 scores than outliers (76.2 vs. 64.5, *p* = 0.007) (Figure 2).

Only 8.3% (25) of KA‐TKA cases fell outside ±2 SD of the control phenotype distribution. Outliers were associated with severe preoperative deformity (>12° varus/valgus), BMI > 35 kg/m^2^ and significantly lower FJS‐12 scores (*p* = 0.01) (Table 5).

**Table 5:** Outlier phenotype characteristics.

Parameter	Outliers (*n* = 25)	Nonoutliers (*n* = 275)	*p* value
Preoperative HKA (°)	−13.2 ± 3.1	−5.8 ± 2.7	<0.001
BMI (kg/m^2^)	34.1 ± 3.8	28.9 ± 3.4	<0.001
FJS‐12	54.3 ± 11.2	70.1 ± 10.8	0.01

Abbreviations: BMI, body mass index; FJS‐12, Forgotten Joint Score‐12; HKA, hip–knee–ankle angle.

To contextualize the postoperative restoration of phenotype, the preoperative alignment of the TKA cohort was analysed against the control distribution. The healthy control cohort established a normative HKA range of −5.2° to +2.8° (defined as mean ± 2 SD). In contrast, the TKA cohort presented with a mean preoperative HKA of −6.8° ± 4.1°, indicating that a substantial majority of patients began with limb alignment outside the normal constitutional range, characteristic of advanced osteoarthritis. The subsequent finding that only 8.3% of patients remained outside this range postoperatively demonstrates a significant normalization of coronal limb alignment through the KA technique, effectively restoring the population's alignment profile to one that closely mirrors the healthy, nonarthritic state.

## DISCUSSION

This study proved that KA in TKA successfully restored constitutional knee phenotypes to population‐specific norms in 91.7% of Iranian with less than 20° of coronal deformity. By respecting the three‐dimensional diversity of coronal alignment (HKA, FMA, TMA) defined by Hirschmann et al. [12], KA‐TKA successfully replicated the phenotypic distribution of healthy Iranian controls (*κ* = 0.92). This explains the observed 18% improvement in Forgotten Joint Scores among phenotype‐matched patients, a measure that is strongly correlated with normal knee kinematics [4]. The restoration of joint line convergence angle (JLCA) to 0.9° ± 0.8° (vs. 0.8° ± 0.9° in controls) confirmed the capacity of KA‐TKA to reestablish dynamic ligament balance, a parameter that is more predictive of outcomes than absolute limb alignment [2].

A critical consideration is how KA successfully restores phenotypes in knees that have undergone significant osteoarthritic deformation. It is essential to clarify that KA aims not to replicate the pathological preoperative phenotype, but to restore the patient's constitutional (prearthritic) phenotype. Osteoarthritis, particularly in varus knees, often manifests as a combination of femoral cartilage wear and more profound tibial wear and bone loss, leading to a pathological increase in varus alignment primarily through a reduction in the mMPTA [12]. The KA technique addresses this composite deformity through a targeted approach: on the femoral side, the component is positioned to resurface the distal and posterior joint lines, thereby largely restoring the native FMA. The primary corrective action occurs on the tibial side. By adjusting the tibial resection to re‐establish a physiological joint line, KA directly corrects the pathological mMPTA that was exacerbated by medial compartment wear [25]. This process effectively ‘neutralizes’ arthritic deformity. Our data, which show a dramatic shift of the population's HKA from a preoperative mean of −6.8° (pathological varus) to a postoperative mean of −1.3° (constitutional varus), visually demonstrate this restorative correction. Therefore, KA acts as a powerful tool to convert the osteoarthritic phenotype back to the native state by respecting the constitutional anatomy of the femur and systematically correcting the acquired tibial deformity.

While MA‐TKA creates nonphysiologic joint lines in 89% of cases [6], KA‐TKA demonstrated superior proprioceptive recovery, evidenced by stair‐climbing efficiency in early rehabilitation [4, 11, 20]. This is consistent with Howell's gait analyses, which show that KA‐TKA patients achieve ascension times 25% faster than their MA‐TKA counterparts due to preservation of collateral ligament isometry [4]. Furthermore, the strong correlation between JLCA normalization and KOOS‐Pain scores (*r* = 0.71) raises the possibility that partial instability, rather than absolute alignment, may be the cause of dissatisfaction.

The strong divergence in retrieval accuracy between females (*κ* = 0.94) and males (*κ* = 0.85) suggests that sexual dimorphism is a critical variable in TKA planning. Iranian females exhibited higher native tibial valgus (41.6% vs. 12.8% in males), necessitating distinct alignment targets. KA‐TKA accommodated these differences through patient‐specific bone resections, whereas MA‐TKA forces neutral alignment, which compromises female kinematics [17, 21]. In this study, 41.6% of females and 12.8% of males showed valgus TMA (*p* < 0.001). JLCA was 1.2° ± 0.8° in the 279 females and 0.9° ± 0.7° in the 171 males (*p* = 0.01). These anatomical realities explain why females who underwent unrestricted KA‐TKA achieved 93.3% restoration in valgus‐dominant phenotypes (177° | 90° | 87°), while historical MA‐TKA data show 30% higher dissatisfaction rates in women [21]. This data shows the important role of gender‐specific limits, when ±5° of tibial varus may be safe for Iranian men but could carry a risk of instability in women with constitutional valgus alignment.

While KA successfully restores native knee phenotypes in over 90% of patients, the 8.3% outlier cases involving severe deformities (>20° varus/valgus) require a managed approach utilizing rKA boundaries, patient‐specific instrumentation or robotic assistance to achieve optimal balance between anatomical restoration and implant longevity, with hybrid techniques or constrained implants reserved for cases with ligamentous insufficiency [1, 15, 30]. However, our recent study has shown that unrestricted KA‐TKA can be as effective in severe cases of deformity as in mild deformities [26].

Our findings indicate a profound ethnic variation in the constitutional alignment of the limb, which necessitates population‐specific KA‐TKA limits. Iranian knees showed 53.7% constitutional varus (HKA < 178°), while this rate was 28.3% in Swiss groups [23]. This study showed a HKA threshold of −3.2° ± 2.1° with good clinical results, which is in contrast to the Danish population, where varus >−2° increased the risk of revision [17, 31]. Neutral HKA has been shown in 38.2% of our patients, similar to Southeast Asia with 31.2% prevalence [9], but far lower than Swiss cohorts (62.1%) [14]. These variations explain why unrestricted KA‐TKA generated 8.3% outliers in Iranians, primarily severe varus (>12°) and obese (BMI > 35) cases, while performing optimally in mild deformities. Consequently, ethnic‐adjusted rKA boundaries are proposed: ±4° HKA for Iranians (vs. ±3° for Europeans), with tibial varus tolerance up to 87° to accommodate prevalent CPAK I phenotypes [17]. Research on this ethnic adjustment during TKA may be possible in the future.

The research contains multiple restrictions that need to be evaluated when making decisions about applying these results to broader populations. The difference in ages between TKA patients at 68.2 years and controls at 28.4 years theoretically creates a possibility of incorrect age‐based limb angle changes, particularly the JLCA, but the constitutional alignment theory supports stable phenotypes. Data from only one surgeon provides technical consistency yet makes the results less applicable to broader populations because standardized PSI protocols reduce implant position errors to 0.7° ± 0.3°. The research uses contralateral limb alignment as a method to estimate prearthritic alignment, but this approach assumes perfect limb symmetry, although research shows only 26% of individuals maintain identical bilateral phenotypes, yet excluding lateral differences above 3° reduces this source of confounding.

## CONCLUSION

This study confirms KA's capacity to restore constitutional knee phenotypes in 91.7% of Iranian patients with less than 20° of coronal deformity, establishing a paradigm‐shifting principle that alignment targets must be population‐specific when a ‘neutral’ HKA is nonphysiologic for 61.8% of Iranians. Furthermore, females require distinct coronal targets and implant morphologies to accommodate tibiofemoral dimorphism.

## AUTHOR CONTRIBUTIONS

**Mohammad Mahdi Sarzaeem**: Conceptualization. **Ali Saeidi, Rasoul Shirmohammadi, Mohammadsajjad Sarzaeem** and **Yashar Shahbaz**: Methodology. **Salar Baghbani, Mohammad Movahedinia, Ali Saeidi, Rasoul Shirmohammadi, Mohammadsajjad Sarzaeem** and **Yashar Shahbaz**: Investigation. **Salar Baghbani, Rasoul Shirmohammadi** and **Mohammad Movahedinia**: Formal analysis. **Salar Baghbani, Rasoul Shirmohammadi** and **Mohammad Movahedinia**: Writing—original draft preparation. **Mohammad Mahdi Sarzaeem**: Resources. **Mohammad Mahdi Sarzaeem**: Writing—review and editing. **Mohammad Mahdi Sarzaeem**: Supervision. All authors reviewed and approved the final version of the manuscript.

## CONFLICT OF INTEREST STATEMENT

The authors declare no conflicts of interest.

## ETHICS STATEMENT

This retrospective cohort study received ethical approval from the institutional review board (IR.SBMU.RETECH.REC.1403.714) and adhered to the Declaration of Helsinki. All participants provided written informed consent.

## Data Availability

Available in response to request to the corresponding author.
